# Reassured on a background of vulnerability - people with microvascular angina 12 months after high-intensity physical exercise program

**DOI:** 10.1080/17482631.2022.2162452

**Published:** 2022-12-28

**Authors:** Ingrid Ølfarnes Røysland, Venke Irene Ueland, Alf Inge Larsen

**Affiliations:** a Department of Public Health, Faculty of Health Sciences, University of Stavanger, Stavanger, Norway; b Department of Caring and Ethics, Faculty of Health Sciences, University of Stavanger, Stavanger, Norway; c Department of Cardiology, Stavanger University Hospital, Stavanger, Norway

**Keywords:** Exercise/physical activity, experiences, heart-health, pain, uncertainty

## Abstract

**Purpose:**

Physical activity is recommended for patients with coronary microvascular dysfunction, however, avoided due to fear about the heart. The aim is to achieve an understanding of the meaning of physical activity one year after participating in a high-intensity exercise training program.

**Method:**

Twelve people were interviewed using a phenomenological hermeneutic approach.

**Results:**

Four themes were formulated and revealed that one year after participating in high-intensity exercise training participants had an awareness of the meaning of the project, their chest pain and daily life: Being reassured, Daily life’s impact on chest pain and continuing doing high-intensity exercise training, A strengthened body and mind, Being part of a group of people with similar problems.

Comprehensive understanding was formulated as “Being reassured according being physically active in a background of vulnerability”.

**Conclusion:**

This study indicates that by going through the high-intensity exercise training program the person regains more unity with the lived body and an unfolding life. A person-centred approach is suggested including an underlying dimension of vulnerability. A lifeworld led care means meeting the patient in their way of relating to the world bodily and existentially. Taking this understanding into consideration will advance the requirements for establishing person-centred care.

## Introduction

Chest pain is one of the most common complaints in medical settings (Safdar et al., 2018). There has been an increase in research related to biological explanations, especially progress in diagnosis of myocardial ischaemia (Kaski et al., 2018). Following Safdar et al. (2018) the coronary microcirculation represents one of the present limits in cardiology research, and it is suggested that more light must be shed on the rising area of coronary microvascular dysfunction. As this condition is hard to visualize directly there are often no signs even if the persons have symptoms like chest pain (Safdar, Ong & Camici, 2018). The medical-technical advances of the last century have created increased possibilities for investigation and treatment. In this regard there is perhaps a possibility for underestimating persons’ experiences caused by illness, due to a tendency to seek objectively measurable disease. Health care systems need to include both.

In accordance with the increased body of research investigating the cause of chest pain and various treatments (Chambers et al., 2015; Suhrs et al., 2018) physical activity has become an important part of rehabilitation programs for cardiac heart disease (Anderson et al., 2016; Dibben et al., 2018; Kaminsky et al., 2019; Khanji et al., 2018). At the same time there are indications of avoidance behaviour related to physical activity (Jonsbu et al., 2011; Nelson & Churilla, 2015).

## Background

Physical activity is defined as “any bodily movement produced by skeletal muscles that results in energy expenditure” (Caspersen et al., 1985, p. 126). Exercise is further a compartment of physical activity and defined as “structured and repetitive and has final or intermediate objective as improvement of, or maintenance of physical fitness” (Caspersen et al., 1985, p. 126). According to Tremblay and Pyke (2019) exercise training is described as perhaps the most physiological and best approach to exploit the adaptive capacity of the coronary vascular bed, and to evoke several functional and structural changes. At the same time, we know that avoidance of doing physical activity in patients with exercise-induced angina pectoris is described in research (Lochbaum et al., 2017) and in addition observed in clinical practice (Jonsbu et al., 2010, 2011; Simonÿ et al., 2017).

A considerable concern in cardiac rehabilitation programs is according to Mezzani et al. (2013) the intensity of aerobic exercise. High-intensity interval training involves repeated periods of activity of short duration (bouts), high to severe or severe to extreme intensity exercise separated by short periods of lower intensity. Further, this has been recommended for improving exercise capacity and has been perceived as being more effective than continuous exercise (Guiraud et al., 2012; Mezzani et al., 2013; Moholdt et al., 2012; Vanhees et al., 2012). However, contradictory results have been presented (Ellingsen et al., 2017). This points to the need for more knowledge in this area.

According to Røysland et al. (2017) patients with angina-like chest pain with no obstructive coronary artery disease who participate in a high-intensity exercise training program undergo a transition described as becoming a more capable person. To our knowledge there is no follow-up on the continuation of the process of training from these patients’ perspective. Patients’ experience one year after participating in a high intensity exercise training program may have considerable potential for giving us more complete understanding from the patient perspective. The aim of the present study therefore was to achieve an understanding of the meaning of physical activity 12 months after taking part in a high-intensity physical exercise program for people living with microvascular angina. Specific focus was directed to what the participants learned and what elements were considered as most helpful.

## Method

This study was part of the SYNDEX study, a larger project. The main objectives were to assess the effect of high-intensity aerobic exercise training on coronary flow reserve, exercise capacity, endothelial function, level of angina and psychological function in individuals with chest pain and no obstructive cardiac arterial disease. However, the coronary flow reserve is the limiting factor for myocardial perfusion when there is no coronary epicardial disease.

For this study of the phenomenon of physical activity in daily life for persons living with microvascular angina, a qualitative approach was chosen as the most suitable. The consolidated criteria for reporting qualitative research (COREQ) checklist (Tong et al., 2007) was used for reporting of research findings (File S1). In the present study the phenomenological hermeneutical approach (Lindseth & Norberg, 2004), inspired by the philosophy of Ricoeur (1976) was used. People have been telling their stories about their experience. According to Ricoeur (1976) the prerequisite for creating knowledge about human experiences is that people communicate their lived experiences, and these are transferred into textual form. By means of a dialectic movement between the whole and the parts, from understanding to explanation and back to understanding, knowledge can be created, and in addition from what the text conveys to what it says (Ricoeur, 1976). The sample selection, data collection, analysis and rigour considerations have been guided by the phenomenological hermeneutical approach.

### Participants and data

Eligible patients were checked according to the inclusion criteria first by a nurse, then by a physician and finally a cardiologist who contacted the patients. Those who met the inclusion criteria were invited to an information meeting where all the health professionals contributing to the project participated.

At this meeting the patients were told about the project and given the opportunity to ask questions. Patients had one week to think through the given information. After agreeing to participate they were contacted again and invited to a medical investigation approximately 7 days after the information meeting. After this investigation they signed of the consent form.

**Figure 1.**
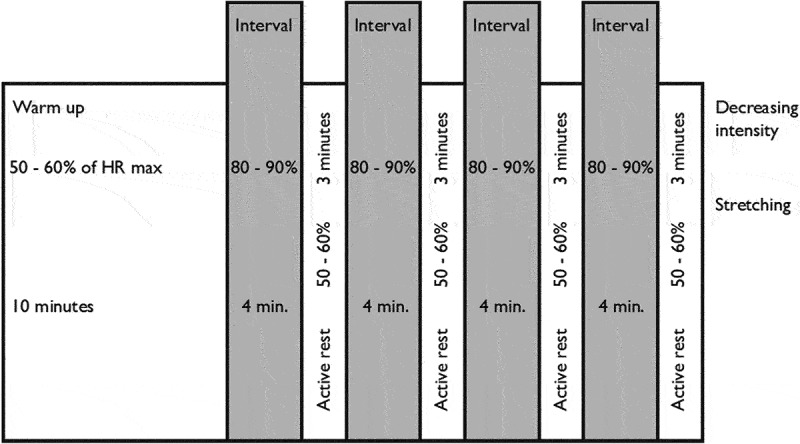
High-intensity aerobic exercise training program, HRmax = maximum heart rate. Frequency and duration: three times per week for 12 weeks.

Twelve participants agreed to participate in an exercise training program (Table I). The results at 12 weeks have been previously reported (Røysland et al., 2017). The patients were now interviewed one year after finishing the program to assess the long-term effects (Figure 1).

**Table I.** Criteria for participating.

Inclusion criteria: outpatients and patients admitted to the hospital with repeated episodes of exercise induced chest pain. normal or near normal coronary angiogram or negative CT coronary angiogram age >18 years able to participate in training groups 3 times a week for twelve weeks Exclusion criteria prior myocardial infarction serious valve disease cancer previous radiation or drug therapy for cancer musculoskeletal problems making exercise training impossible ongoing serious inflammatory disease intra cardiac devices presumed insufficient acoustic windows because of severe emphysema or gross overweight atrial fibrillation participation in other ongoing studies contrast allergy contraindication to adenosine infusion vasospastic angina

In Table II the demographic data are presented.

**Table II.** Characteristics of participants.

Characteristics	
**Gender**	
Female	7
Male	5
**Age**	
40-45	1
46-50	1
51-55	-
56-60	2
61-65	3
66-70	4
71-75	1
**Marital status**	
Married	10
Divorced	1
Cohabitant	1
**Children**	
1-2	7
3-4	5
**Educational attainment**	
Vocational certificate	8
College/university up to 4 years	2
College/university more than 4 years	2
**Employment condition**	
Working full time	4
Working part time	1
Sick leave	2
Disability pension	2 (1 part time because of an injury)
Retirement pension	4
**Chest pain for:**	
4–6 months	1
7–12 months	1
1–2 years	2
2–4 years	5
4–6 years	3
**Smoking -** Daily	1
**Drinking alcohol -** Daily	-

### Ethical approval

Ethical approval was obtained from the regional ethical committee (2013/98/REK vest). The investigation conformed to the principles outlined in the Declaration of Helsinki. Prior to the inclusion in the study all participants provided informed consent. (ClinicalTrials.gov Identifier: NCT02905630).

### Data collection and analysis

Several tests were performed before starting the exercise training: Coronary Flow Reserve (CFR), echocardiography, flow-dependent vasodilation, blood tests and 24-hour heart rhythm monitoring. To assess exercise capacity, electrocardiography changes, angina, blood pressure and arrhythmia at baseline and at 12-week follow-up, ergo spirometry during a maximal cardiopulmonary stress test on a treadmill was accomplished. Unstable patients with arrhythmia or pathological blood pressure during the test were excluded.

The data analysed and presented in the current article consist of individual interviews one year after the exercise training program was finished.

### Data collection

The interviews took place between November 2014–June 2015. The location was an undisturbed room in the same building where the participants had been doing the exercise training. The interviews lasted for approximately one hour and were conducted by the first author. The interviews were open-ended (Mishler, 1991). The participants were encouraged to speak in greater depth about what it was like to be physically active during the year after participating in the project. They were invited to talk in their own words and then asked follow-up questions as: “Could you expand on that?” and “Could you give more examples from daily life?” (Mishler, 1991). The interviews were audio-taped and transcribed verbatim. In the transcripts non-verbal expression such as laughter, crying, and pauses were noted (Mishler, 1991).

As described by Lindseth and Norberg (2004) the transcription is part of the phenomenological hermeneutical method of interpretation. Choices are always made in relation to methodological approaches (Davidson, 2009). The relationship between recordings and the events that they provide information about must be considered, as well as the recordings and transcripts (Duranti, 2006). The transcriptions in this study, as in all studies, have been carried out within certain cultural, historical, and biographical frames.

### Data analysis

A three-step method developed by Lindseth and Norberg (2004), inspired by Ricoeur (1976) was used when the text was analysed: consisting of naïve interpretation, structural analysis, and comprehensive understanding.

*Naïve interpretation*. From an initial reading of the text a naïve interpretation is formulated. To gain a naïve understanding of what it is like to be physically active for people living with no obstructive coronary artery disease one year after participating in a high-intensity exercise training, each interview was repeatedly listened to and read as open-mindedly as possible. The first assumption guided and provided the ideas for the subsequent analysis.

*Structural analysis*. The authors first systematically examined the data set. The purpose was to express the essential meaning contained in everyday words to gain a deeper understanding of the interview content before dividing the text into meaning units. In each interview the units were subsequently compared for similarities and differences. Then the condensed meaning units were abstracted and organized before formulating subthemes and themes.

*Comprehensive understanding*. The authors formulated a comprehensive understanding of the text as a whole after reflecting on the naïve reading, comparing the themes with the literature and finding that the initial interpretation and subsequent structural analysis validated each other. The authors reflected on pre-understanding, initial naïve interpretation and structural analysis while taking the different levels of data into consideration.

Table III presents an example of the three-step thematic analysis to obtain meaning units, condensed meaning units, and subthemes. We chose to present direct quotations from the participants to preserve authenticity of results. Variation is exemplified therein. These are examples of lived experiences of the meaning of physical activity for people with no obstructive artery disease. The core commonalities or variations are described within these examples.

**Table III.** Meaning units, condensed meaning units, and subthemes, examples.

Meaning Unit	Condensed Meaning Unit	Subtheme
What I find difficult sometimes is when I, I have a job where I travel. It will be long evenings. And so, it’s okay when you go by bicycle to work, then, I have to cycle home anyway, you have to go home anyway. But it’s very hard to walk away to the gym when I work late at night, and a little tired, and yes. And then when we’re travelling, because then it can be hectic. Then you never get time to exercise like that. So, then it’s a bit like it requires some effort when we get home. But it goes quite well, in a way because it is motivation. Because I know that the training keeps it in check. If I do not exercise, I fall back. I can fall back. Can you say, so, so that’s actually motivation? And it is, it is not more than we did here. It’s the 40 minutes, so it’s fast.	Being physical active in a way which fits in with daily life	Reflecting on daily life’s impact on being physical active
I walked (on a treadmill in the gym) for a while then, yes, August, a little then. And yes, that was nice, it was nice. Nice when you arrived. But that’s enough, I probably do not like such a form of training where you have to drive away. For me, cage is a bit far from everything. Yes, so then I think rather: No, take on your jacket and shoes and go out. Because I feel, I have always felt, that it gets a little fussy to go on something at that and that time. Yes, so that’s why I am slipping away.		

## Methodological Considerations

The aim of the present study was to achieve an understanding of the meaning of physical activity 12 months after taking part in a high-intensity physical exercise program for people living with microvascular angina. Specific focus was directed to what the participants learned and what elements were considered most helpful.

The findings are based on rich data from in-depth interviews with 12 Norwegian people diagnosed with microvascular angina, one year after participating in a high-intensity exercise training program. The criteria of credibility pertain to the researcher’s ability to describe the way one became familiar with the context where the interviews were conducted. The first author participated in the information meeting before the project started. She also partly participated when the tests were performed. In addition, she spoke to the health personnel, which gave a nuanced insight into the structure and environment of the unit. The credibility of the investigation was strengthened by discussions in meetings with the co-authors. The verification strategies of Morse et al. (2002) were used throughout the process. We focused on establishing agreement between the different sets of themes and preliminary conclusions during the analysis, achieving this by taking the levels of data into consideration along with our reflections on pre-understanding.

The intention has been to present the research process in a manner that makes it possible for the reader to follow the different steps, as well as the logic of and reasons posterior to the concluding remarks. The whole truth can according to Lindseth and Norberg (2004) never fully be understood. Nevertheless, we searched for possible meanings in a continuous process from naïve reading and structural analysis through to comprehensive understanding. Knowledge, claims, findings, and interpretations in the data are also part of confirming trustworthiness, and the described theoretical foundation, findings, and data analysis are aimed at fulfilling the criterion of conformability.

## Results

### Naïve interpretation

The naïve assumption suggests that physical activity is practiced against a background of vulnerability, and at the same time reassurance after a thorough examination of the heart and participating in the high-intensity exercise training.

### The first structural analysis

We asked the participants where their activity took place one year after participating in the project, and in addition how they did it. The reason was to achieve an understanding of exercise training contexts for the purpose of situational framing.

Various arenas were described by the respondents. Some had continued the training on a treadmill at home or at a gym, running and cycling outside in the natural environment, and some described no training, but still being physically active in daily life.

The participants described different levels of physical activity. Some had continued the same level of exercise as when participating in the project and had even expanded that. Others were still living with a lot of limitations related to physical activities for different reasons.

The participants’ vulnerability and reassurance were revealed in the naïve interpretation along with arenas for physical activity. The variety of activities and experiences of chest pain duration point to the need to know more about meanings of being physically active. The analysis of some of the narrative reveals the interpretive possibilities of the text.

### Second structural analysis: themes

In the thematic analysis, focus was directed towards the meaning of physical activity and high-intensity exercise training in life for people with no obstructive heart disease, against a background of vulnerability. A specific focus was directed to what the participants learned and what elements were considered as most helpful. Four themes and ten subthemes were formulated, and revealed that one year after participating in high-intensity exercise training participants had an awareness of the meaning of the project, their chest pain and daily life.

Being reassured, Daily life’s impact on chest pain and continuing doing high-intensity exercise training, A strengthened body and mind, Being part of a group of people with similar problems. An overview of the subthemes and themes is shown in Table IV.

**Table IV.** Overview of Subthemes and Themes.

Subtheme	Theme
Reflecting on the projects influence of being physical active	
● Investigation of the heart ● Experience of doing high intensity exercise training	Being reassured.
Reflecting on having learned to interpret bodily sings while doing physical activity	
Reflecting on what they had learned according warming up before hard exercises	
Reflecting on daily life’s impact on chest pain	Daily lives impact on chest pain and continuing doing high-intensity exercise training
Reflecting on daily life’s impact on being physical active/continuing with high intensity exercise training	
Reflecting on other health issues restricting high intensity exercise training.	
Experiencing strengthened body and mind while doing physical activity	A strengthened body and mind
Experiencing absence or reduced chest pain while continuing doing high intensity exercise training	
Experiencing absence or reduced arrythmia/extra systoles when doing high intensity exercise training	
Reflecting on the meaning of having been part of a group with people with similar problems	Been part of a group with people with similar problems

*Being reassured*. This theme reflects the meaning for the people participating in the project one year after termination. Many of them had been told they had no heart disease when contacting the health care system for consultation when experiencing chest pain. Being asked to participate in this project, assuming they had a microvascular coronary dysfunction, meant being taken seriously. The thorough examination of the heart further reassured them that they did not have more serious heart diseases.

*I’m not worse. I’m not. Um maybe a little better, but maybe it has something to do with the fact that I know what it is, I think so.*

Further the experience of doing high intensity exercise training at an individual level supervised by highly experienced health professionals gave was described as reassuring. The location of the training, near the hospital, with defibrillators on the wall, was also mentioned as comforting. The experience of what they felt in their bodies when doing this exercise made them interpret what they felt in another way.

*I have felt so confident in this plan I had here, in what I endured. Absolutely fantastic, yes … Oh yes, it was of course that you had, that you had the people around you who knew this. And if anything were to have happened, glory, and no, I would never have done that. Had never done that. No, I had never understood once that it was something I should do.*

The participants appreciated learning how to start exercise training in a proper way. During the project they had experienced the value of warming up before the high-intensity training. This reduced or removed the chest pain while doing exercise training.

*Then I completely abandoned the idea that there could be something wrong with my heart, because, because they drove us pretty hard down there and then I thought that then … But again, I think it has to do with warming up before you work hard. It helps. I think that if they had put me right on that treadmill under full pressure right away, then something would have stopped.*

They were reflecting on having learned to interpret bodily signs while doing physical activity. They were not uncertain in the same way as before joining the project. However, they still described a vulnerability connected with physical activity.

*Daily life’s impact on chest pain and continuing doing high-intensity exercise training*. The participants considered different reasons for their chest pain in daily life. Some described great pressure, like children’s diseases, spouses’ diseases, and spouses’ challenges as addictions. Others talked about challenging job situations. They reflected on what impact this had on their chest pain.

*So, I wish in a way that it was only these pains I had to deal with, but it is never so in life. For life is so complicated. But I have not given up yet in any case. That is, considering the personal situation. I haven’t, but it has sometimes come close. I just have to say, it’s not that easy.*

Further, the participants reflected on this as a background for continuing the physical activity at the same level as they did for twelve weeks when exercising in the project. All participants followed the schedule for training three times a week for all 12 weeks. Some were still strictly following this habit.

*I still do the training. I got the treadmill for Christmas, brand new and very nice. And it has gone very smoothly. So much of the time I have followed the program.*

However, others found it challenging to continue being physically active at the same level as when being part of the project.

*And then I was good for a while, but then it dropped off a bit, and then there comes a holiday and such, so, so it has not been, well yes. But I go for daily walks and such.*

The participants were also reflecting on other health issues restricting high intensity exercise training. Examples were having pain like “frozen shoulder”, or in the knees and legs.

*A strengthened body and mind when doing high-intensity exercise training over time*. The participants described bodily symptoms before, during and after participating in the exercise training in the project. They described chest pain as being the same, being reduced, and some did not have any chest pain while continuing doing exercise training after participating in the project.

*No, I think something happened when I last trained with [the project], I do not know if it is mental or what it is. But then, I got it explained that the blood vessels expand, and all and this with the heart, which is, that you’ve got to train them up, simply.*

Some who had arrhythmia, extrasystoles when doing high intensity exercise training before participating in the project now experienced reduced symptoms or absence of these.

There seemed to be a desire to learn to interpret bodily symptoms when doing high intensity exercise training. It was described as important to be supervised by the health professionals when doing the exercise training, and being reassured when interpreting the bodily symptoms.

*I calm down a bit. So, I’ll become much better at feeling it, or, yes. Take it all into account. Yes, if there is no more to go on. Then it’s not wrong. And, em, and it was a big task, and when it went well, yes. So I’m comfortable. So, it was a good test. Actually, it has gone very well, in fact. I think so. So, a year and a half ago I was barely able to walk up to the fourth floor at work, from the basement, you might say. At least it was very painful, and today I am running up.*

*Been part of a group of people with similar problems*. The participants reflected on feeling alone with their problems before being asked to participate in the project. Some highly valued the people in the group who they were exercising together with. They experienced a fellowship with people with similar problems and found exercising together with them as precious. Others felt it was like training alone even if they were together with people with the same problems. However, none of the participants had kept in touch with the other participants after completing the project.

*Yes, it is, it went better, because first there was a bit of such talk, and they were motivated and ready to train a bit, and it’s clear. You came in now and there was a bit of small talk before you went home again, but, but you heard the talk, you stood right next to each other, and you saw that everyone got better results. And as I said, you saw that they survived, the whole gang, and hopefully they were better, at least in retrospect, so you saw it worked for most everybody. And that they, yes, there was no problem. At least there was no one in my group who had to break off anything, at least I think so. No. No. So, then everyone managed to complete the program without getting too much pain.*

### Comprehensive understanding

Now the interpretation can move one step further. The meaning of physical activity in life 12 months after taking part in a high-intensity physical activity program among people living with microvascular angina was formulated in the themes: Awareness of participating in the project as a background for being reassured, the impact of daily life and other health issues on chest pain and doing high-intensity exercise training, experiencing a strengthened body and mind when doing high-intensity exercise training over time, and the meaning of having been part of a group of people with similar problems. Out of this a comprehensive understanding was formulated: Being reassured according being physically active in a background of vulnerability.

Being physically active when having microvascular angina disease one year after taking part in a high-intensity exercise training program is seen as improving health and life, but at the same time challenging. The participants were reassured that it is safe for them to do high-intensity exercise training. They experienced this by participating in the project. The high-intensity exercise training did not provoke heart disease. On the contrary their health improved by doing this exercise. But even though they were reassured their symptoms were still in the background. There lies an existential dimension therein such as vulnerability. In addition, it was a challenge to continue doing high-intensity exercises in daily life.

This is highlighted by the French philosopher Maurice Merleau-Ponty’s Phenomenology of perceptions (Merleau-Ponty, 2012). He explains both the pre-reflexive character that our original relation with the world has, as well as the understanding that our body develops within a context provided by the world. The participants reflected about “physical activity”, “high-intensity exercise training”, “improving health”, and “habits”.

Merleau-Ponty’s explanation of habit (Merleau-Ponty, 2012) expresses a kind of body knowledge that cannot completely be understood by neurological processes. He uses concepts like those of the lived or own body, and of lived space in order to highlight what exists between subject and the world. The participants talked about being reassured, and as a subject being able to do high-intensity exercise training in the world. Merleau-Ponty (2012) addresses issues and provides analysis that is crucial for an understanding of the true complexity of consciousness and cognition. Within this he places the contemporary situation. For the participants in the present study the contemporary situation can be deduced from a thorough investigation of the heart and having the opportunity to participate in high-intensity exercise training simultaneously as having a dialogue with experienced health professionals.

The participants discussed their motivation for continuing their physical activity after finishing the project. Following Merleau-Ponty (2012) we can speak of a motivation of the part of the world, although not of a necessity. This is because the response is not mechanical or determined. Habit bears a direct relation to this form of dialogue between environment and subject (Merleau-Ponty, 2012). The participants were reflecting about finding space for being physically active in their daily life.

Merleau-Ponty (2012) claims that human beings ordinarily experience themselves as a unity of subject and object. A person’s embodiment as a unity can be broken when the person experiences illness. As we see it, the seamless unity might be broken when experiencing uncertainty related to chest pain. A form of immediate being-in-the -world can be lost (Merleau-Ponty, 2012). The participants who made high-intensity exercise training a new habit in daily life experienced improved health. It seems that being conscious about what the heart could handle and that the pain is not due to angina, soothes them. It is as if they get a new embodied anchoring, that the unit is strengthened, and they can to a greater extent live their lives.

According to Merleau-Ponty (2012) the lived human body relates to a space that also is lived. The experience of participating in the high-intensity exercise training relates to the emergence of a new horizon. For the participants that means they can do exercise training on the background of having gone through the exercise training in the project.

## Discussion

The aim of the article was to achieve an understanding of the meaning 12 months after taking part in a high-intensity physical exercise training program for people living with microvascular angina. Specific focus was directed to what the participants learned and what elements were considered most helpful. The comprehensive understanding was formulated as ”Being reassured according being physically active in a background of vulnerability”. Key issues from comprehensive understanding in relation to previous research form subheadings in the following discussion.

### Vulnerability

The participants were reassured after being thoroughly investigated for heart disease and participating in the high-intensity exercise training program. They experienced that it was safe for them to do high-intensity exercise training. However, their previously or present symptoms like chest pain were in the background. This makes them vulnerable. From a phenomenological perspective, vulnerability is related to our embodied state in particular. This according to Carel (2009) is the distinction between objective and subjective vulnerability. Avoiding physical activity is an example of objective risk. By avoiding physical activity people are exposed to what is described as objective vulnerability. Our response to illness varies and there is no necessary relationship between what Carel (2009) describes as illness and feeling subjective vulnerability.

Gjengedal et al. (2013) claim that vulnerability is an existential phenomenon. The participants expressed previous fears that high-intensity exercise training would provoke a heart attack and even death. Following Gjengedal et al. (2013), as vulnerability is an existential phenomenon that belongs to the basic conditions of life, our vulnerability is to be accepted and considered rather than fought. The participants in the present study have to accept and consider their situation on the background of their signs and symptoms. Their signs are here understood as the information they got from the investigation (Edwards, 2008), and symptoms as the subjective experienced illness (Dodd et al., 2001). Gjengedal et al. (2013) claim the degree of vulnerability will vary depending on the situation and cultural context, making vulnerability a contextual phenomenon.

The participants in the present study described the context and situation of the high-intensity exercise training they participated in a year ago as reassuring. Our identities are only complete through our commitment to others, according to Purcell (2013). The high-intensity exercise training was located close to the hospital, with defibrillators on the wall and experienced health professionals as supervisors. Patients with the same issues were doing the same exercise training. The relationship in the group, as well as no one being unwell during the exercise training, was in addition referred to as reassuring.

### Health

The findings from this article indicate that living with chest pain affects health in several different ways. Health is particularly affected when there is uncertainty about what the chest pain means, how serious and how life-threatening the pain is. In the phenomenology of the body, our access to the world, living one’s everyday life, goes through the lived body.

Dahlberg and Segesten (2010, p. 62) define health as a balanced feeling of freedom and vulnerability that constitutes a person’s wellbeing and capacity to pursue minor and major life projects that are valued by the person. In the present study this influences being reassured while being physically active. Parallels can be drawn with the results of Røysland and Friberg (2016) where the participants with unexplained chest pain balanced between existential uncertainty and certainty when being physically active. The uncertainty was related to doing physical activity which was seen by the participants as a threat to health and life. However, at the same time, physical activity was seen as improving health and life. If we take into account Dahlberg and Segesten (2010) definition of health as a balanced feeling of freedom and vulnerability, then the life situations of the participants are freer, and the vulnerability has receded into the background one year after taken part in a high-intensity exercise training.

In the present study the participants were thoroughly examined for any organic cause of the chest pain. Chest pain as their subjective experience indicated that something was wrong with the heart. They had a need for more information to be able to confidently assess whether it was safe and if it improved their health to do high-intensity exercise training. This is in line with Røysland et al. (2013) who revealed unmet information needs of people with unexplained chest pain. There was a need for more knowledge on how to do physical activity in a safe way. Health professionals must be prepared to meet both verbalized and more implicitly posed information needs.

Marcum (2008) claims that physicians as well as other health professionals mostly have implemented an understanding that disease is conceptualized exclusively in terms of the physical body. Some participants in the present study reflected on earlier experiences where their subjective experience was discounted and viewed as unreliable or irrelevant by the health professionals. The importance of seeing the person as a unity of body and mind is fronted. Merleau-Ponty (2012) claims the patient is situated in a lifeworld as an embodied subject. The body orients the patient to the world around them by means of their symptoms like chest pain. This can be understood as the patient’s existential concern being associated with the experience of the sensation in the body when exercising. They observed their bodies, their heartbeat, extrasystoles and chest pain and reasoned with themselves as how to understand this in relation to their bodies.

According to Eriksson (2001), health is linked to acting and certain ways of acting. Beliefs related to having or acquiring a serious heart disease were reformulated by participants, and there was reflection about high-intensity exercise training being good for the heart. The participants expressed that they were able to do high-intensity activity in daily life, having in mind warming up before starting. The example here is shovelling snow. This points to some kind of freedom against the background of being vulnerable.

The results revealed that daily life was affected by the ability to make choices. Patients experienced a new opportunity to live their daily life. This is in line with Nussbaum (1988) who talks about illness as a disruption in life. Some patients experienced chest pain as a causal link for giving up exercising alone. Participating in the exercise training program broke the link and helped them one year afterwards to exercise autonomy.

The participants discussed the value of the experience of having been doing high-intensity exercise training three times a week for 12 weeks a year ago. The participants had the experience as an emerging horizon. This is incorporated as a subject inserted into a world that provokes certain questions or problems that must be resolved.

### Person-Centered approach

The participants in the present study reflected on being taken seriously and listened to when participating in the exercise training in the project. By listening to the patient’s story, human resources and opportunities can be identified. This can be a platform for a health plan as a partnership. For the participants in this study this has been a ground for further plans of exercising.

A person-centred approach is suggested to support patients with microvascular angina. Different approaches and labels are used for person-centred care, and there is no commonly agreed definition (Edvardsson et al., 2008). A person-centred approach means according to Ekman (2014) and McCormack and McCance (2006) including the patient, and sometimes people close to the patient. This approach can encourage health professionals to see beyond task-oriented information, as it is a relational ontology behind patient-centred care (Ekman et al., 2011).

Where health professionals anchor the person-centred approach in what is described as an ethical perspective (Ekman et al., 2011; Ricoeur, 1990), there is an obligation to recognize and acknowledge the fragility of self and coherence in life. This emphasizes according to Ekman et al. (2011) the importance of seeing the person as worthy and capable in partnership with health professionals. Central values such as mutual understanding and respect for the patient’s self-esteem and will, are the foundation in the relationship between the patient and the health professional (Ekman, 2014).

Within the relationship between the participants and the health professionals was a reciprocity and a focus on the patients’ individual problems. In addition, an openness towards different ways of expressing questions was experienced. Health professionals have to be aware of the everyday language ambiguity in the patient’s narratives (Ekman, 2014). This implies a need for guidance and education in putting person-centred care into practice.

The patient is seen as an active partner when health professionals have a person-centred approach. In the present study the participants talk about how they balance their activity and exercise in daily life. According Merleau-Ponty (2012) the healthy person is able to come and go from habitual to the actual. There is “another self” that has already sided with the world and is previously open to define its aspect and synchronize with them.

Merleau-Ponty (2012) explains that habitual behaviour arises on the basis of a set of situations and responses that despite not being identical, constitute a community of meaning. The participants in the present study well exemplified this, which indicates a significant human potential. According to Merleau-Ponty (2012) this is possible because the body “understands” the situation in which it must act. The power persons have of dilating of being in the world, or altering our existence by incorporating new instruments is expressed by a habit (Merleau-Ponty, 2012).

## Conclusion

The results reveal the value of meeting the patients with their individual needs and for the patients to be thoroughly examined when having symptoms like chest pain. Especially there is a value for the patient of experiencing their body sensations going through a high-intensity exercise training. One year after participating in a high-intensity exercise training for 12 weeks the patients were reassured that it was safe to be physically active in daily life, also on a level of high-intensity. For some this meant incorporating a new habit in daily life, and this implies improved health.

Chest pain may cause a break in the embodied lifeworld, so that the immediate unfolding of life is prevented. This study indicates that by going through the high-intensity exercise training program the person regains more unity with the lived body.

We argue that this must include an underlying dimension of vulnerability to operate on an appropriate level of explanatory depths. Hopefully this study contributes aspects and nuances related to going through the high-intensity exercise training. A person-centred approach is suggested to support in explicating questions and beliefs. This is also a ground for further plans of activity for the patients with coronary microvascular dysfunction. A lifeworld led care means meeting the patient in their way of relating to the world bodily and existentially. One suggestion for future research is interventions with larger number of participants to understand critical aspects more fully.
